# Optimiser la gestion de la douleur chronique : explorer la contribution essentielle du personnel infirmier en soins primaires

**DOI:** 10.1080/24740527.2024.2394207

**Published:** 2024-10-07

**Authors:** Andréanne Bernier, Marie-Eve Poitras, Anaïs Lacasse

**Affiliations:** a Département des sciences de la santé, Université du Québec en Abitibi-Témiscamingue, UQAT, Rouyn-Noranda, Québec, Canada; b Département de médecine familiale et de médecine d’urgence, Université de Sherbrooke, Campus Saguenay, Sherbrooke, Québec, Canada; c Département des sciences de la santé, UQAT, Rouyn-Noranda, Québec, Canada

**Keywords:** douleur chronique, rôle infirmier, activités infirmières, soins primaires, *Chronic Care Model*, Chronic pain, nurse role, nursing activities, primary care, *Chronic Care Model*

## Abstract

**Introduction:**

L’expertise infirmière en soins primaires est cruciale pour répondre aux besoins des patients vivant avec une maladie chronique. Au Canada, une personne sur cinq vit avec de la de douleur chronique (DC), créant ainsi un fardeau socioéconomique majeur. Cependant, le rôle du personnel infirmier en soins primaires en DC reste sous-développé.

**Objectifs:**

Cette revue narrative analyse l’adéquation du récent Plan d’action québécois en DC avec les modèles de soins reconnus pour la gestion des maladies chroniques et examine le rôle potentiel du personnel infirmier dans la mise en œuvre de ce plan.

**Méthode:**

La synthèse de la littérature a été menée à partir de diverses bases de données (CINAHL, PubMed) et sources documentaires en français et en anglais. Les résultats ont été interprétés à travers le prisme du *Chronic Care Model*, un cadre reconnu pour améliorer les soins.

**Résultats:**

Bien que le Plan d’action québécois en DC soit aligné sur les objectifs visés, sa mise en œuvre devra surmonter divers défis. Les constats indiquent des opportunités efficaces dans la gestion de la DC, comme le montrent des études sur la pratique infirmière en soins primaires. Elles révèlent que le personnel infirmier exerce déjà des activités de gestion des maladies chroniques applicables à la DC.

**Conclusion:**

Il est important de reconnaître l’apport de l’expertise infirmière en soins primaires pour réévaluer les modèles d’organisation des soins, promouvoir un partage de responsabilités fondé sur les données probantes, et soutenir la recherche future ainsi que l’innovation clinique dans la gestion de la DC.

## Abstract (en)

**Introduction:** Primary care nursing expertise is crucial in meeting the needs of patients living with chronic disease. In Canada, one in five individuals lives with chronic pain (CP), creating a major socioeconomic burden. However, the role of primary care nursing in CP remains underdeveloped.**Objectives:** This narrative review analyzes the alignment of the recent Quebec Action Plan on CP with recognized chronic disease management models and examines the potential role of nurses in implementing this plan.**Method:** A literature synthesis was conducted using various databases (CINAHL, PubMed) and documentary sources in both French and English. Results were interpreted through the lens of the Chronic Care Model, a recognized framework for improving chronic diseases care.**Results:** Although the Quebec Action Plan on CP aligns with its intended goals, its implementation will need to overcome various challenges. Findings indicate effective professional opportunities in CP management, as evidenced in studies of primary care nursing practice. They reveal that nursing staff already engage in chronic disease management activities applicable to CP.**Conclusion:** We recognize the contribution of primary care nursing expertise is crucial for reassessing care organization models, promoting evidence-based shared responsibilities, and supporting future research and clinical innovation in CP management.

## Introduction

Depuis des décennies, le système de santé du Québec, comme ailleurs dans le monde, cherche à répondre aux besoins croissants de soins liés au vieillissement de la population et à l’augmentation significative du fardeau des maladies chroniques.^1,2^ On sait qu’une organisation efficace des soins primaires, favorisée par une meilleure accessibilité et une coordination accrue entre les professionnels de la santé, permet d’améliorer la santé de la population tout en optimisant les ressources.^3^ Plusieurs études reconnaissent que l’utilisation du plein potentiel de la pratique infirmière peut contribuer à l’efficacité et à l’efficience des systèmes de soins de santé, notamment pour mieux répondre aux besoins complexes des personnes soignées ainsi que d’assurer la coordination, l’accessibilité et la prestation de soins sécuritaires et de qualité.^4–7^

Au Canada, la douleur chronique (DC) touche une personne sur cinq.^8^ Son fardeau économique en termes de soins de santé et de perte de productivité atteint 38.3–40.4 millions de $ CAN par année.^9^ Par ailleurs, aux États-Unis il a été estimé que le fardeau économique de la DC est plus important que celui des maladies cardiaques, du diabète ou du cancer.^10^ Force de constater, les travaux découlant du Plan d’action canadien en matière de DC stipulent qu’à l’heure actuelle, les personnes vivant avec de la DC ne sont pas toujours traitées de façon optimale.^9,11^ Dans la même foulée au Québec, le nouveau Plan d’action en DC du ministère de la Santé et des Services sociaux (MSSS) souligne l’importance de passer à l’action pour une meilleure gestion de la DC. Il est entre autres suggéré d’établir une trajectoire de soins qui s’étend sur un continuum en incluant les trois paliers de services, débutant par les soins primaires, mettant de l’avant une organization des soins qui repose sur la collaboration interprofessionnelle et qui est centrée sur les personnes.^12^

On peut ainsi se demander comment la profession infirmière pourrait contribuer au Plan d’action québécois en DC^12^ et améliorer la qualité et l’accessibilité des soins. Le rôle infirmier en soins primaires dans la gestion de la DC est cependant peu développé, tant au Québec, au Canada que dans le monde.^13–15^ Une façon de mieux situer ce rôle est de le positionner grâce aux modèles d’organization des soins reconnus pour la gestion des maladies chroniques. Une revue de la littérature récente s’avérait donc pertinente pour répondre à deux questions de recherche: 1) Dans quelle mesure le Plan d’action québécois en DC est-il en adéquation avec les modèles d’organization des soins reconnus? et 2) Comment le personnel infirmier peut-il contribuer à la mise en œuvre de ce plan d’action dans le contexte actuel du système de santé? La présente revue narrative visait donc à analyser, à travers le prisme de modèles d’organisation des soins reconnus pour la gestion des maladies chroniques, la contribution potentielle du personnel infirmier dans la mise en œuvre du Plan d’action québécois en DC.

## Méthodologie

Une revue narrative a été menée pour nous permettre de répondre à nos deux questions de recherche. Cette revue inclut un examen rigoureux de la littérature récente, sans toutefois prétendre avoir identifié toutes les études sur le sujet, et sans évaluation de la qualité des études. Les résultats ont été synthétisés de manière narrative.^16,17^ Bien que les revues systématiques soient généralement privilégiées pour la pratique fondée sur les données probantes, nos questions n’étaient pas suffisamment spécifiques pour justifier une méthodologie systématique. De plus, l’intention était de trouver de la littérature pour soutenir notre analyse du Plan d’action québécois en DC, plutôt que d’évaluer la portée ou la qualité des études disponibles.

Pour assurer une compréhension approfondie du sujet, cette revue narrative, réalisée en septembre 2023, a adopté une stratégie de recherche sans restriction de temps à partir des bases de données CINAHL (EBSCOhost) et MEDLINE (PubMed), couvrant ainsi les domaines des sciences infirmières et de la santé. Les critères d’inclusion comprenaient tous types d’études (quantitatives, qualitatives, mixtes) publiées en anglais ou en français. Ces études devaient porter sur le rôle et les activités du personnel infirmier dans la gestion des maladies chroniques et de la DC, ainsi que celles portant le contexte de la prise en charge de la DC en contexte de soins primaires. Les études portant sur la pratique infirmière avancée ou le rôle infirmier en clinique spécialisée de la douleur ont été exclues afin de tenter d’isoler la plus-value de la pratique infirmière dite généraliste.^18^ Les mots-clés ont été choisis en fonction de l’expérience et de l’expertise de l’équipe de recherche pour maximiser la portée de la recherche, incluant des termes tels que *nurse, nurse role, nursing activities, primary care nurse, primary care, family medicine, general practice* et *chronic pain*. Les résultats de la recherche ont été gérés dans un logiciel de gestion de références (EndNote®). Une recension de la littérature grise a également été incluse dans notre analyse. En effet, la revue narrative offre une flexibilité méthodologique qui permet d’inclure un large éventail de sources de données nécessaires à l’analyse d’un plan d’action complexe, dont la portée et les implications sont multidimensionnelles.^16^ En intégrant divers types de littérature, cette méthode facilite la compréhension des interactions complexes entre les parties prenantes du système de santé, ainsi qu’une exploration approfondie des contextes historiques, politiques et sociaux influençant l’élaboration du plan d’action.^16^ Les sources consultées comprenaient les sites, entre autres, de l’Ordre des infirmières et infirmiers du Québec (OIIQ), de l’Association des infirmières et infirmiers du Canada (AIIC), du ministère de Santé et des Services sociaux du Québec (MSSS), de l’Institut national en santé publique du Québec (INESSS), de l’*International Association for Study of Pain* (IASP), ainsi que les répertoires de mémoires et de thèses. Les textes de loi ont également été consultés à partir des sites gouvernementaux. Les titres et résumés des documents ont été examinés par AB pour une première sélection basée sur les critères d’inclusion. AL et MEP ont supervisé ce processus. Étant donné la nature de cette revue narrative visant à synthétiser l’état actuel des connaissances utiles à l’analsze du Plan d’action québécois en DC, aucune évaluation de la qualité des articles n’a été effectuée.^17^ Les résultats ont été inclus s’ils mettaient en lumière la contribution du personnel infirmier en soins primaires, que ce soit sur la santé physique, sociale, cognitive ou mentale, les compétences d’autogestion, ou les résultats mesurés ou autorapportés, ainsi que les résultats sur le système de santé, comme la charge de travail, l’utilisation des services et les coûts. Pour répondre à nos deux questions de recherche, nous avons interprété les résultats en utilisant le cadre du *Chronic Care Model*,^19^ en classifiant les données extraites selon ses différentes composantes. La *Scale for the Assessment of Narrative Review Articles* a guidé la rédaction de cet article.^20^

## Assises théoriques

Il existe un nombre considérable de modèles d’organisation des soins, y compris des approches telles que le *Chronic Care Model*,^19^ le *Stepped Care 2.0*,^21^ le *Patient-Centered Medical Home*^22,23^ ou le *Collaborative Care Model*,^24,25^ chacun offrant des cadres uniques pour améliorer l’organisation des soins. Pour nourrir notre démarche réflexive, le *Chronic Care Model*^19^ a été retenu comme cadre pour structurer l’ensemble des angles d’analsze en raison de son approche spécifique de la gestion des maladies chroniques, mettant l’accent sur une prise en charge coordonnée, intégrée et préventive. Par sa vision systémique et globale, ce cadre conceptuel permet de guider l’orientation des meilleures pratiques pour la gestion optimale des maladies chroniques dans un système de santé,^26,27^ facilitant ainsi leur mise en œuvre. Développé aux États-Unis par le *MacColl Center for Health Care Innovation*,^19^ il est régulièrement utilisé dans l’analyse et la restructuration d’activité clinico-administratives en maladies chroniques au Québec.^28^

Brièvement, les six composantes de ce modèle sont: 1) l’organisation du système de santé; 2) la communauté, les ressources et les politiques; 3) le soutien à l’autogestion; 4) la conception du système de prestation des services; 5) le soutien à la prise de décision; et 6) le système d’information clinique.^26,27^ Les deux premières composantes concernent plus largement « le système de santé entourant les services de santé », soit: 1) l’organisation des soins de santé qui réfère à une organisation offrant des soins sécuritaires et de haute qualité par le soutien à l’amélioration continue à tous les niveaux (de la gouvernance aux prestataires de soins) et 2) la communauté, les ressources et les politiques qui réfère à la mobilisation de toutes les ressources disponibles (collaboration intersectorielle avec les organisations communautaires et politiques) pour soutenir le système de santé afin de répondre aux besoins des personnes et favoriser leur participation active.^27^ Les quatre autres composantes du modèle touchent plus spécifiquement « la prestation des soins auprès des personnes soignées ». Le soutien à l’autogestion place la personne au centre des soins et englobe les activités d’éducation thérapeutique et les activités de développement des compétences d’autogestion pour aider les personnes à mieux vivre au quotidien avec leur condition (autonomisation).^27^ La conception du système de prestation des services réfère aux pratiques collaboratives, à la coordination des soins, à la continuité des soins, à la gestion de cas et aux soins culturellement sécuritaires.^27^ Le soutien à la prise de décision réfère à des soins basés sur des données probantes et orientées sur les besoins des personnes en rendant accessibles des outils de dépistage et d’évaluation, des outils cliniques et des lignes directrices à jour ainsi qu’à la formation, au perfectionnement des prestataires de soins et au partage d’expertise (soins primaires et soins spécialisés).^27^ Finalement, le système d’information clinique réfère aux ressources technologiques pour soutenir la communication efficace et le partage des informations permettant le suivi des personnes vivant avec une maladie chronique.^27^ C’est donc basé sur ces deux angles d’analyse (les services de santé et la prestation des soins), que le *Chronic Care Model* a guidé la réflexion sur la publication récente du Plan d’action en DC du MSSS du Québec^12^ et l’implication possible du personnel infirmier en soins primaires auprès des personnes vivant avec de la DC.

## Organisation des soins pour la gestion des maladies chroniques telles que la douleur chronique

Avant de se plonger dans l’analyse des orientations québécoises en matière de gestion de la DC selon le *Chronic Care Model*, il importe de comprendre les différents modèles d’organisation de soins en maladies chroniques et la trajectoire de soins pour la patientèle vivant avec de la DC.

Tout d’abord, la hiérarchisation des services est la base de l’organisation des soins auprès des personnes vivant avec une maladie chronique,^29^ incluant la DC.^30^ La hiérarchisation implique une intégration des services de soins de première (soins primaires), de deuxième et de troisième ligne tant au niveau local, régional que national.^29,30^ Pour assurer une offre de service efficace et efficiente, les services du continuum de soins doivent donc être complémentaires et coordonnés, et les trajectoires de soins doivent être clarifiées et appuyées par des mécanismes de référence limitant les délais et les complications pour les personnes.^29,30^ Au cœur de cette hiérarchisation, la communication des informations sur les soins de santé des personnes est un élément phare.^29,30^

Plus précisément, dans une approche de « soins par paliers », les soins primaires sont responsables d’offrir une variété de soins qui portent sur la promotion de la santé, la prévention des maladies et la gestion des maladies chroniques (incluant l’autonomisation et le soutien au développement des compétences d’autogestion des personnes).^3^ Les principales cibles d’activités des soins primaires en ce qui a trait à la gestion des maladies chroniques sont l’évaluation complète de la condition de santé et du bien-être de la personne, le diagnostic et l’initiation du traitement.^29,30^ Par la suite, les prestataires de soins primaires assurent le suivi et l’accompagnement de la personne, le soutien à l’autogestion, l’identification des objectifs de santé et l’élaboration d’un plan de soins.^29,30^ Selon le *Kaiser Permanente* aux États-Unis et le *National Health Service* en Angleterre, ce niveau de soins devrait pouvoir répondre aux besoins de 70 à 80% des personnes vivant avec une maladie chronique.^31^

À l’heure actuelle, le réseau de soins primaires offrant des soins dédiés à la gestion des maladies chroniques est formé d’une gamme d’organisations de services publics et privés. Le modèle privilégié d’organisation publique de soins primaires au Québec qui assume principalement cette responsabilité est le Groupe de médecine familiale (GMF) et le Groupe de médecine familiale universitaire (GMF-U); 382 regroupements (au 30 juin 2023).^32^ Un GMF est composé d’un groupe de médecins de famille travaillant avec d’autres professionnels de la santé issus par exemple des soins infirmiers, du travail social, de la pharmacie, etc.,^33^ et qui peut être un milieu d’enseignement pour la médecine (GMF-U). De façon parallèle, il existe des services par le biais de cliniques médicales privées. Il est également possible de recevoir certains services en matière de maladies chroniques par le centre local de services communautaires (CLSC) sous forme de programmes de soutien,^34^ sans nécessairement être accompagnés de services médicaux.^35^ Enfin, plusieurs cliniques publiques d’infirmières praticiennes spécialisées (IPS) voient le jour.^36^

Les services spécialisés et ultraspécialisés (2^e^ et 3^e^ ligne), requérant des expertises professionnelles ainsi que des infrastructures et des technologies de pointe, devraient donc être utilisés pour venir en support aux besoins des prestataires de soins primaires selon la complexité de la condition des personnes (qui représente respectivement les besoins de 15% et 5% des personnes vivant avec une maladie chronique).^29,30^ Ces milieux sont aussi responsables des activités d’enseignement et de formation dans le réseau de la santé.^29,30^ Enfin, il importe de comprendre que compte tenu du caractère chronique, ponctué de périodes d’exacerbation aiguë de la maladie et de détérioration de la condition de santé, la trajectoire de soins des personnes vivant avec une maladie chronique comme la DC n’est pas exclusivement linéaire.

En matière de services spécialisés en DC au Québec, la 2^e^ et 3^e^ ligne est assurée respectivement par des centres régionaux en gestion de la douleur chronique à travers la province, chacun associé à l’un des quatre centres d’expertise en gestion de la douleur chronique (CEGDC) de chacun des réseaux universitaires intégrés de santé et de services sociaux (RUISSS) de la province. En 2^e^ ligne, on retrouve des professionnels issus de diverses expertises dans le domaine de la médecine et de la réadaptation situés dans les hôpitaux et les cliniques externes (ex. anesthésistes, physiatres, psychologues, ergothérapeutes, etc.) pour faire des évaluations plus approfondies pour déterminer les causes sous-jacentes de la DC et offrir une gamme de traitements plus spécialisés. Pour la 3^e^ ligne, situés dans les grands centres urbains (deux à Montréal, un à Québec et un à Sherbrooke), les CEGDC offrent une expertise multidisciplinaire en douleur pour élaborer des plans de traitement personnalisés et complets pour les patients souffrant de douleur chronique grave et complexe visant à améliorer leur qualité de vie.^37^

Un autre élément phare des modèles d’organisation de soins en maladie chronique est la collaboration interprofessionnelle.^29,30^ Dans la littérature et les documents, « multidisciplinarité » et « interdisciplinarité » sont souvent utilisées de manière interchangeable. Toutefois, il est plus juste de parler de « collaboration interprofessionnelle » qui réfère plutôt à un continuum de collaboration entre les prestataires de soins.^38^ Selon la complexité de la situation de soins, la personne peut être amenée à travailler avec plusieurs prestataires de soins en parallèle (multidisciplinaire) ou à établir un plan de soins coordonné et partagé avec une équipe travaillant ensemble (interdisciplinarité) pour répondre aux besoins de la personne qui sont multiples et qui touchent tous les aspects de sa vie quotidienne avec la maladie.^38^ D’ailleurs, les personnes vivant avec de la DC sont reconnues parmi les conditions chroniques pour avoir un des plus grands niveaux de complexité de soins.^39^ En grande majorité, les personnes vivant avec une maladie chronique comme la DC devraient pouvoir compter en soins primaires sur un médecin de famille et le personnel infirmier pour assurer leur suivi, mais les pharmaciens, les nutritionnistes, les physiothérapeutes, les travailleurs sociaux peuvent être interpelés selon les besoins biopsychosociaux.^40,41^

## Pourquoi un nouveau plan d’action québécois en DC?

Au début des années 2000, l’Agence d’évaluation des technologies et des modes d’intervention en santé (AETMIS)^30^ soulignait le besoin criant pour le système de santé de développer une trajectoire de soins optimale pour la gestion de la DC, notamment en s’inspirant de l’approche d’offre de services par paliers (hiérarchique) de façon intégrée et coordonnée. Il était aussi précisé que l’interdisciplinarité est essentielle à la gestion de la douleur et que le personnel infirmier pouvait contribuer davantage à la gestion de celle-ci. Or, après plus de 15 ans, le MSSS^12^ publiait des orientations et lignes directrices pour soutenir les milieux de soins dans l’optimisation des soins en DC (nouveau plan d’action). Force de constater que des besoins persistent dans la gestion de la DC, le ministère souligne le manque d’accessibilité des services en DC, le manque de coordination entre les paliers (soins primaires vs cliniques spécialisées) et le besoin d’une approche centrée sur la personne.^12^ Au niveau des soins primaires, tels que les GMF, il est mentionné que les personnes vivant avec de la DC ont peu accès au personnel infirmier, en travail social ou en pharmacie^12^ et sont majoritairement seulement pris en charge par des médecins de famille.^9,42^ Enfin, les centres secondaires de 2^e^ ligne sont déployés de façon variable sur le territoire occasionnant plusieurs références vers la 3^e^ ligne des centres surspécialisés, sans trajectoire de soins établis pour assurer un suivi en soins primaires.^12^

### En théorie, mais en pratique ?

C’est à partir d’ici qu’il est intéressant d’analyser l’adéquation du Plan d’action québécois en DC à travers les différentes composantes du *Chronic Care Model* afin de voir son potentiel pour l’amélioration des soins pour les personnes vivant avec de la DC. Brièvement, le Tableau 1 présente les trois principaux axes d’interventions du Plan d’action québécois en DC ainsi que les grands objectifs associés, en plus de faire le parallèle avec les six composantes du *Chronic Care Model*. En revanche, les grands objectifs du Plan d’action se divisent en plusieurs objectifs spécifiques qui peuvent être catégorisés dans plus d’une composante du *Chronic Care Model*, puisqu’elles sont interdépendantes et que le concept de gestion d’une maladie chronique est dynamique et multifactoriel. En théorie, notre analyse nous permet de voir que le Plan d’action québécois en DC semble être aligné avec les recommandations présentées précédemment et être cohérent avec un modèle reconnu pour la gestion exemplaire des maladies chroniques. Le Plan d’action en DC se positionne judicieusement pour promouvoir des soins centrés sur le patient, une coordination efficace des soins, l’autogestion, l’utilisation des technologies en santé et l’intégration du système de santé apprenant.

**Tableau 1.** Plan d’Action en douleur chronique 2021–2026 classifié avec les composantes du *Chronic Care Model*.

Plan d’action en douleur chronique 2021–2026^12^	*Chronic care model*^26,27^
**Axe sur l’accessibilité**	
OBJECTIF 1: Améliorer la prise en charge de la douleur chronique à tous les niveaux de soins en favorisant une approche interdisciplinaire et biopsychosociale, et ce, pour l’ensemble de la population pédiatrique, adolescente, adulte et gériatrique OBJECTIF 2: Améliorer les mécanismes d’accès entre les différents niveaux de soins et de services	L’organisation du système de soins de santéLa conception du système de prestation des services
**Axe sur le rôle du patient**	
OBJECTIF: Favoriser l’autonomie et la prise de décision partagée entre le patient et le professionnel de la santé	La communauté, les ressources et les politiques Le soutien à l’autogestion
**Axe sur le transfert de connaissances**	
OBJECTIF: Soutenir les cliniciens (médecins et professionnels de la santé) dans les meilleures approches en gestion de la douleur et du travail en interdisciplinarité par des activités de formation, de mentorat et de consultation	La communauté, les ressources et les politiques Le soutien à la prise de décision
**Axe sur l’évaluation et l’amélioration de la qualité**	
OBJECTIF: Instaurer une culture de l’amélioration continue et de la pratique réflexive sur le plan de la pratique clinique et organisationnelle en gestion de la douleur chronique, ancrée sur les besoins des patients	L’organisation du système de soins de santéLe système d’information clinique
**Axe sur la gouvernance**	
OBJECTIF: Assurer un suivi au plan stratégique	L’organisation du système de soins de santéLe système d’information clinique

**Figure 1.**
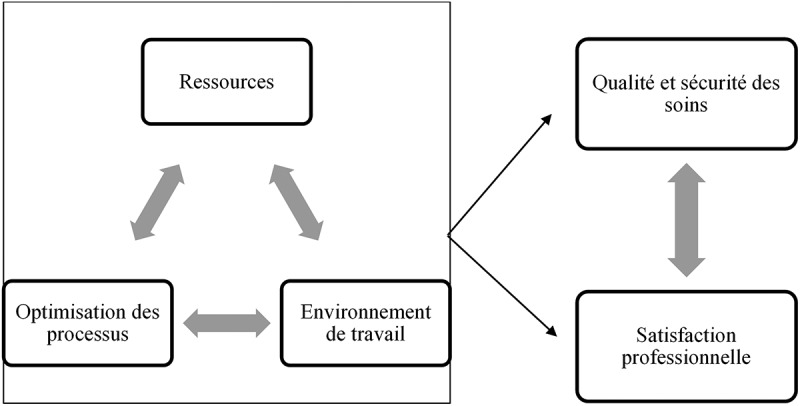
Modèle d’organisation des soins adapté de Dubois el al.^43^

En pratique, il importe cependant d’analyser les moyens ou les actions à prendre pour atteindre ces objectifs pour en évaluer la faisabilité, dans la mesure des ressources de l’organisation. En ce sens, issus des sciences infirmières, Dubois et al.^43^ proposent trois concepts à considérer comme des leviers de transformation de l’organisation des soins pour assurer la qualité et la sécurité des soins, en plus de favoriser la satisfaction et le bien-être professionnel, soit: 1) l’acquisition, le déploiement et le maintien des ressources (ex. caractéristiques propres de l’organisation des effectifs/composition des équipes et caractéristiques des personnes soignées), 2) l’environnement de travail (incluant la capacité d’innovation en utilisant les technologies pour répondre aux besoins de santé de manière efficiente et l’environnement en soutien à la pratique professionnelle), et 3) l’optimisation des processus de soins (éventail des responsabilités et des fonctions assumées par le prestataire de soins pour réponse aux besoins de santé) (Figure 1). Ce modèle, complémentaire au *Chronic Care Model*, retient notre attention puisqu’il permet « d’illustrer les liens entre les leviers et d’analyser comment la structure des ressources et des processus contribue à l’obtention de résultats spécifiques dans un contexte donné » (traduction libre),^43^ et donc, d’opérationnaliser les moyens et les actions possibles pour mettre en œuvre un plan d’action. Le Tableau 2 apparaît donc pertinent pour présenter les obstacles les plus identifiés dans la littérature du domaine de la DC et classifiés selon le modèle de Dubois et al.^43^ afin d’approfondir notre analyse.

**Tableau 2.** Obstacles documentés à la gestion de la douleur chronique en soins primaires^9,11,41,72,73,81^ classifiés selon le modèle de Dubois et al.^43^

Acquisition, déploiement et maintien des ressources
Diversité de la composition des équipes des prestataires de soins primaires Charge de travail élevée en soins primaires en lien avec l’adéquation les effectifs en place
Optimisation des processus de soins
Difficulté à établir le diagnostic (subjectivité de la DC et multifactorielle) Formation et développement professionnel en spécifique DC limité Échelles validées, outils cliniques et lignes directrices peu connus et/ou peu utilisés Besoin d’outil pour le dépistage et pour déterminer le niveau de pratique collaborative requise (profil biopsychosocial et complexité de la condition; comorbidités) Peu de gestionnaires de cas pour les personnes ayant des besoins complexes Connaissances limitées à l’égard des traitements pharmacologiques et de leurs effets indésirables ainsi que la gestion de la polypharmacie Recours aux approches « non pharmacologiques » limité et/ou peuvent être couteuses (en particulier si absence d’assurance santé adéquate).
Environnement de travail
Approche médico-centrique prédominante et besoin de favoriser le travail en équipe (formation en collaboration interprofessionnelle) Accès limité aux spécialistes (davantage en milieu rural ou défavorisé), souvent centralisé et longues listes d’attente pour les services spécialisés Absence de systèmes d’information partagés et problèmes de coordination avec les prestataires en pratique privés et les autres paliers de services

En somme, en comparant la liste des grands objectifs du Plan d’action en DC (Tableau 1) et la liste d’obstacles (Tableau 2), on peut constater que presque tous les grands objectifs et les obstacles se recoupent. C’est donc dire que le Plan d’action doit trouver des stratégies de mise en œuvre pour déconstruire les modèles de soins traditionnels en place, valoriser la place centrale de la personne soignée, maximiser le travail de collaboration interprofessionnelle, arrimer le partenariat communautaire et potentialiser l’utilisation des technologies.^44^ Bref, bien que ce Plan d’action québécois en DC soit porteur de bonnes pratiques reconnues, la mobilisation des moyens et des actions à prendre pour atteindre ses objectifs devra être importante compte tenu des ressources déjà limitées du système de santé au risque de compromettre la faisabilité de ce plan d’action. C’est donc dans cette perspective qu’on peut se questionner sur l’apport d’une masse critique de nos intervenants de santé qu’est le personnel infirmier.

## La contribution infirmière à la gestion de la douleur chronique

L’étendue de la pratique infirmière consiste en un ensemble de fonctions et de responsabilités professionnelles légalement confié à la profession infirmière en lien avec les compétences, les connaissances et les habiletés pour les exercer.^45,46^ Spécifiquement au Québec, l’adoption de la Loi 90 et la Loi 21 a accordé 17 activités réservées au champ d’exercice infirmier, lui conférant l’un des plus vastes champs d’exercice professionnels autonomes pour contribuer aux services de santé.^47^ Plus précisément, l’activité réservée « effectuer le suivi infirmier des personnes présentant des problèmes de santé complexes » positionne le personnel infirmier comme un pilier pour assurer la continuité des soins auprès des personnes à risque de complications, qui nécessitent des soins coordonnés avec différents prestataires de soins et/ou vivant avec une maladie chronique nécessitant un suivi longitudinal.^47^ Concrètement, il peut évaluer les besoins biopsychosociaux des personnes, dépister des situations à risque, décider d’initier des mesures diagnostiques et thérapeutiques ainsi que d’administrer et d’ajuster des médicaments selon une ordonnance, communiquer des résultats de santé, planifier un congé, faire de l’éducation en matière de santé, assurer le suivi téléphonique et orienter les personnes soignées vers les ressources adéquates.^47^

L’évaluation de la contribution de la profession infirmière à la qualité, à l’efficience et la performance du système de santé (*system outcomes*) est nécessaire pour démontrer l’importance de la main-d’œuvre infirmière et pour éclairer les décideurs, les gestionnaires et les prestataires de soins concernant la mise en œuvre de la pratique infirmière.^7,48^ Or, de nombreuses études ont démontré l’efficacité des interventions infirmières dans la gestion des maladies chroniques,^49–52^ notamment dans la DC.^53–56^ En effet, lorsque les personnes parviennent à mieux gérer leurs maladies chroniques, elles ont plus de chances d’améliorer leur condition de santé et d’adopter de meilleures habitudes de vie, ce qui conduit à une utilisation plus efficace des ressources de santé.^7,49^ De plus, étant donné leur champ d’exercice, le personnel infirmier est l’un des rares prestataires de soins à favoriser l’intégration et la coordination des soins physiques et psychologiq qui sont très prévalents chez les personnes vivant une multimorbidité (co-occurrence de plusieurs maladies chroniques).^57^ L’effectif infirmier (*workforce*) représente également le type de prestataire de soins le plus présent dans les milieux de soins primaires,^58,59^ en plus de représenter un rapport coût-efficacité avantageux pour les systèmes de santé.^7,52^ Les études soulignent aussi la satisfaction élevée des personnes soignées et de leur famille pour les soins reçus, un accès accru aux services de santé et une meilleure perception de la santé générale (déclaré par les patients).^4,60,61^ Au Québec, on dénombre plus de 800 infirmières et infirmiers œuvrant dans un GMF.

Afin de mesurer cette performance, la pratique infirmière s’opérationnalise par la mise en œuvre d’activités de soins (actions) dans la pratique clinique.^62^ Tandis que l’étendue de la pratique infirmière pleinement déployée représente l’occupation maximale du champ d’exercice professionnel par la mise en œuvre complète des activités pour lesquelles le personnel infirmier a été formé et est autorisé à exercer (« *full nursing potential* »),^63^ l’étendue *effective* de la pratique infirmière fait référence à l’éventail des activités, des fonctions et des responsabilités réellement déployées par le personnel infirmier.^45,46^

Grâce à l’approche systémique du *Chronic Care Model*, la revue systématique de Reynolds et al.^64^ a identifié les activités en soins primaires ayant le plus d’effets positifs pour les personnes soignées, les prestataires de soins et le système de santé dans 157 études. Les composantes contenant ces activités (dont plusieurs réalisables par le personnel infirmier) étaient le *soutien à l’autogestion* (45,8%), la *conception du système de prestation des services* (22,6%), le *soutien à la prise de décision* (21,3%) et le *système d’information clinique* (8,9%).^64^ Plus précisément encore, la revue de Dufour^65^ visait à recenser les activités infirmières qui contribuent à la gestion optimale des maladies chroniques en contexte de soins primaires. La revue a permis, entre autres, d’identifier les activités infirmières en gestion de maladies chroniques en soins primaires présentant des résultats significativement favorables pour la santé ou pour l’utilisation des services chez les personnes vivant avec au moins une maladie chronique dans trois composantes majeures du *Chronic Care Model*, soit 1) le soutien à l’autogestion, 2) la conception du système de prestation des services et 3) le soutien à la prise de décision.^65^

## Et si l’infirmière en Groupe de médecine familiale faisait partie de la solution pour la gestion de la DC?

Au Québec, les maladies chroniques les plus couramment prises en charge par le personnel infirmier en soins primaires sont le diabète (98%), l’hypertension artérielle (96%), la dyslipidémie (83%), l’obésité (58%), la dépression/l’anxiété (39%), la maladie pulmonaire obstructivechronique (31%), l’hypo/hyperthyroïdisme (29%), l’asthme (19%), les maladies cardiovasculaires (19%) et l’arthrite (13%).^66^ Considérant qu’il a été démontré qu’il existe une forte prévalence de DC chez les personnes vivant avec ces maladies chroniques,^67–69^ c’est donc dire que les personnes vivant avec de la DC sont déjà en contact avec le personnel infirmier en soins primaires au Québec. Toutefois, dans l’étude de Bergeron et al.^13^ sur les pratiques en matière de gestion de la DC dans les GMF au Québec, 56% du personnel infirmier (n = 53) a rapporté n’être aucunement impliqué dans la gestion de la DC. Les trois activités infirmières en DC les plus fréquemment réalisées ne l’étaient qu’entre 54% et 62% et l’évaluation de la douleur arrivait au septième rang.^13^ Pour cause, le principal obstacle identifié était la méconnaissance des activités infirmières possibles pour soulager la DC à 72%.^70^

En s’appuyant sur les résultats de Dufour,^65^ trois composantes du *Chronic Care Model* apparaissent pertinentes et transférables pour décrire les éléments en faveur d’un rôle infirmier en DC contribuant à l’efficacité du système de santé à l’intérieur du Plan d’action québécois en DC. Dans la composante système de prestation des services, il y a l’omniprésence de la profession infirmière dans les différentes sphères du système de la santé qui représente un positionnement stratégique pour dépister les personnes qui vivent de la douleur, pour évaluer la condition de santé de celles-ci ainsi que pour les guider vers les approches qui visent à soulager la douleur et évaluer leur efficacité.^71^ En effet, des études ont ressorti que le personnel infirmier jouait un rôle significativement positif en DC dans la collaboration interprofessionnelle à travers les activités d’évaluation de la condition de santé, la continuité des soins et les activités de gestion de cas.^53–56^ Puis, pour la composante soutien à l’autogestion, il y a le fait que les personnes vivant avec de la DC ont accès à du soutien pour apprendre à gérer leur condition souvent en 2^e^ et 3^e^ ligne, alors que cela pourrait être offert par des prestataires en soins primaires, dont principalement le personnel infirmier.^72,73^ Pourtant, les interventions en soutien à l’autogestion sont fortement recommandées pour ces personnes puisqu’elles ont de meilleurs résultats de santé à long terme comme la réduction de l’intensité de leur douleur et de l’impact fonctionnel ainsi que l’amélioration de leur qualité de vie.^74–77^ Dès lors, plusieurs revues de la littérature permettent d’affirmer que les interventions infirmières en matière de soutien à l’autogestion auprès des personnes vivant avec de la DC sont efficaces pour augmenter le sentiment d’auto-efficacité de celles-ci.^50,78,79^ Enfin, dans la composante soutien à la prise de décision, plusieurs études sont d’avis que le personnel infirmier pourrait favoriser une meilleure utilisation de la pharmacothérapie en DC et réduire l’utilisation des opioïdes à long terme.^54,55,80–82^

Ultimement, pour situer le rôle potentiel du personnel infirmier dans la mise en œuvre du Plan d’action québécois en DC, rappelons également que leur rôle professionnel se définit comme « une fonction assumée par l’infirmière, modulée par des normes professionnelles, un cadre législatif, un champ d’exercice et un système social ».^62^ Ainsi, pour faciliter une vue d’ensemble de la proposition, le Tableau 3 juxtapose certains objectifs du Plan d’action québécois en DC aux activités infirmières reconnues efficaces pour le système de santé en gestion des maladies chroniques selon le *Chronic Care Model*,^65^ ainsi qu’au champ d’exercice infirmier québécois.^47^ Selon Lukewich et al.,^14^ la mise en lumière des activités transversales de la pratique infirmière en maladies chroniques a un grand potentiel facilitant la transférabilité de la démarche de soins auprès des personnes vivant avec de la DC. En effet, cette a mis en lumière que le revue narrative personnel infirmier réalise déjà des activités en gestion des maladies chroniques qui sont transférables au contexte de la DC où l’évaluation en est l’activité centrale (ex. évaluer la condition de santé, la douleur et ses répercussions, évaluer les besoins pour guider les personnes vers les ressources et les approches qui visent à soulager la douleur et évaluer leur efficacité).

**Tableau 3.** Cohérence du rôle potentiel du personnel infirmier en soins primaires dans le cadre du Plan d’action en douleur chronique québécois.

Plan d’action québécois en DC^12^	Activités infirmières efficaces en soins primaires selon le *Chronic Care Model*^65^	Champ d’exercice infirmier^47^
Axe sur l’accessibilité	Composante *conception du système de prestation des services* Évaluation individualisée: Réfère à **l’évaluation globale du patient et à l’identification des besoins et des risques** de façon à partir desquelles est élaboré un **plan de soins adapté** au patient et **partagé** à **l’ensemble des professionnels** de l’équipe Gestion de cas: Englobe les activités où l’infirmière assure la **coordination des soins d’un patient avec les autres professionnels** et autres structures de soins. Comporte un volet de suivi clinique du patient, afin d’ajuster l’intensité des soins et services en fonction de l’évolution de ses besoins Continuité: La continuité réfère à la **relation entre un intervenant** et le patient et aux aspects de communication et de lien de confiance (rôle pivot), à la disponibilité et à l’utilisation des antécédents du patient afin **de planifier des soins personnalisés et/ou à** l’importance d’une **dispensation de soins au moment opportun et adaptée aux besoins** changeants du patient (suivi en présentiel ou téléphonique). Composante *soutien à la prise de décision* Englobe les **outils d’aide à la décision et protocoles** utilisés par l’infirmière pour prescrire des traitements et des tests diagnostiques	**Évaluer la condition physique et mentale** d’une personne symptomatique **Exercer une surveillance** clinique de la condition des personnes dont l’état de santé présente des risques **Initier des mesures diagnostiques et thérapeutiques**, selon une ordonnance **Administrer et ajuster des médicaments** ou d’autres substances, lorsqu’ils font l’objet d’une ordonnance **Effectuer le suivi** infirmier des personnes présentant **des problèmes de santé complexes**
OBJECTIF: Améliorer la prise en charge de la douleur chronique à tous les niveaux de soins en favorisant une approche interdisciplinaire et biopsychosociale		
Objectifs spécifiques: **Améliorer l’évaluation** du patient de façon à ce que le diagnostic et **le traitement de la douleur chronique puissent être établis** adéquatement, dans le respect des valeurs et des besoins des patients Établir une **offre de service interdisciplinaire** pour la gestion de la douleur chronique **à tous les paliers de soins et de services** Mettre en place **la fonction de gestion de cas** en douleur chronique ayant pour rôle d’intégrer et de coordonner les interventions de l’équipe interdisciplinaire intra et interétablissement		
Axe sur le rôle du patient	Composante *soutien à l’autogestion* Englobe les **activités éducatives** qui soutiennent la capacité de l’individu à **gérer ses symptômes, son traitement ou sa médication**, les **conséquences physiques, psychologiques et psychosociales** en lien avec sa maladie chronique. Inclut fréquemment des **stratégies d’élaboration d’objectifs et de planification des actions** avec le patient.	
OBJECTIF: Favoriser l’autonomie et la prise de décision partagée entre le patient et le professionnel de la santé		
Objectifs spécifiques: **Outiller la personne** souffrant de douleur chronique **en matière d’autogestion** appropriée à sa culture, à son genre, à son âge et à sa condition de santé **Améliorer les connaissances** dans la population, l’entourage et les proches aidants quant à la douleur chronique et à sa gestion		

## Limites

Ce travail de synthèse non systématique présente certaines limites. Tout d’abord, la recherche a été effectuée dans deux bases de données, ce qui pourrait avoir restreint la gamme d’articles examinés. Cependant, cette recherche a permis de recenser une documentation riche, décrivant en profondeur les composantes, les résultats et les défis de la mise en œuvre du rôle infirmier en soins primaires dans le cadre du Plan d’action québécois en DC. Visant à faire valoir la contribution de l’expertise infirmière, notre revue narrative, davantage pragmatique, est sujette à un biais dans les conclusions tirées, où les études avec des résultats positifs ou significatifs sont plus susceptibles d’être incluses. Malgré cela, l’utilisation structurée et transversale du *Chronic Care Model* pour l’extraction et l’analyse des données a permis d’identifier rigoureusement les opportunités pour la pratique infirmière. En revanche, notre revue narrative n’offre qu’un aperçu succinct des facteurs influençant la pratique professionnelle et l’organisation des soins. Enfin, d’autres éléments doivent être pris en compte dans l’interprétation de nos conclusions, tels que la variabilité des contextes en soins primaires, les besoins des personnes soignées vivant avec des comorbidités, et la collaboration interprofessionnelle.

## Conclusion

En somme, la gestion des maladies chroniques demeure un défi pour les systèmes de santé. L’enrichissement de l’actuelle réflexion sur le Plan d’action québécois en DC à partir du *Chronic Care Model* a permis de prendre conscience des multiples enjeux que représente la mise en œuvre d’un plan d’action dans le système de santé. Ainsi, cette analyse a permis de documenter et de cibler, à l’égard de quelles composantes du système de santé, la pratique infirmière peut contribuer à son efficacité et son efficience. En effet, cette analyse a mis en lumière que le personnel infirmier réalise déjà des activités en gestion des maladies chroniques qui sont transférables au contexte de la DC. Cette étape était primordiale pour influencer un partage des rôles et des responsabilités entre les prestataires de soins impliqués dans la gestion de la DC afin de repenser des modèles d’organisation de soins plus innovants et à la hauteur des expertises de chacun, mais surtout basés sur les évidences favorisant la santé des personnes soignées. Dans ce continuum, les prestataires de soins primaires ont un rôle important à jouer puisqu’ils sont les piliers de cette approche d’organisation des soins. Le personnel infirmier en soins primaires représente un acteur clé pour la mise en œuvre de ce Plan d’action québécois en DC et il sera important d’investir des ressources pour faciliter leur positionnement.

## Supplementary Material

Manuscript Bernier et al_CanJPain_R2_Clean.docx

## Financement

La réalisation de la présente revue narrative n’a pas fait l’objet d’un financement spécifique, outre les frais de diffusion des résultats qui couverts via les fonds de fonctionnement de la Chaire de recherche institutionnelle en épidémiologie de la douleur chronique de l’Université du Québec en Abitibi-Témiscamingue dirigée par AL.
